# Au-Co Alloy Nanoparticles Supported on ZrO_2_ as an Efficient Photocatalyst for the Deoxygenation of Styrene Oxide

**DOI:** 10.3390/nano15130957

**Published:** 2025-06-20

**Authors:** Hashini T. Abeyrathna, Chamodi L. Fernando Thibiripalage, Huai Yong Zhu, Eric R. Waclawik

**Affiliations:** School of Chemistry and Physics, Queensland University of Technology, Brisbane, QLD 4001, Australia; w.abeyrathna@qut.edu.au (H.T.A.); chamodi.thibiripalage@qut.edu.au (C.L.F.T.); hy.zhu@qut.edu.au (H.Y.Z.)

**Keywords:** photocatalysis, Au-Co alloy nanoparticles, epoxide deoxygenation, characterisation, synthesis

## Abstract

Epoxide deoxygenation by photocatalysis was explored using Au-Co alloy nanoparticles supported on ZrO_2_ under visible light irradiation. The active metals were deposited on commercial monoclinic ZrO_2_ by chemical impregnation to achieve controlled mass ratios of gold and cobalt in the alloy nanoparticles. The characterisation of the alloy nanoparticles confirmed the technique produced an average particle size of 4.50 ± 0.29 nm. Catalysts containing pure 3% Au and different Au-Co metal ratios attached to the ZrO_2_ induced the deoxygenation of styrene oxide in an isopropanol solvent medium. Only 20 mg of pure Au/ZrO_2_ catalyst gave a 99% yield of styrene at an 80 °C temperature within 16 h under visible light irradiation (400–800 nm). Au-Co/ZrO_2_ catalysts generally induced conversion to styrene under the same conditions below 60 °C. Above 60 °C, a new reaction pathway was observed to favour a different product over Au-Co/ZrO_2_, which was identified as styrene glycol. This study developed a new approach to the synthesis of styrene glycol, a molecule that has many useful applications in the chemical and polymer industries. Surface-enhanced Raman spectroscopic (SERS) studies and electron paramagnetic resonance spectroscopic (EPR) studies identified changes in the reaction mechanism and pathway upon increasing the cobalt molar ratio in the Au-Co alloy catalysts.

## 1. Introduction

The conversion of epoxides to corresponding alkene products is an important reaction in organic and biological chemistry. In organic synthesis, it is frequently used in the protection–deprotection cycle of carbon–carbon double-bond synthesis [1,2]. This transformation also occurs in biological systems during the production of vitamin K in the vitamin K cycle [1,3]. Existing methods for carrying out this reaction require excess amounts of toxic and expensive reagents (such as iodides, silane compounds, low-valent metals, and phosphines) to achieve decent yields [2,4]. They generally operate with low atom efficiency, low catalytic activity, moisture, and air-sensitive reaction conditions, and require tedious workup procedures [1,2,5]. Identifying a sustainable methodology to conduct this reaction therefore represents a valuable and significant endeavour.

Photocatalysis uses light to achieve chemical transformation, potentially making it a pollution-free synthesis method [6,7]. Heterogeneous photocatalysts can harvest light energy to achieve these chemical transformations and have the useful advantage of the relatively easy separation of the catalyst from the product stream. Noble metal plasmonic nanoparticles such as Cu, Au, and Ag when incorporated with semiconductors, are often highly efficient photocatalysts because the attached noble metal can increase charge separation and thereby suppress electron–hole recombination. Alloy nanoparticles containing a noble metal can also be expected to engender efficient photocatalysis by the same mechanism. Where there is a synergy between the alloyed metals, a commonly observed trend is the further enhancement of catalytic activity and the creation of new properties. In catalysis, the addition of a second metal can modify surface chemistry and create new active sites for reactions to occur, while still promoting charge separation for improved photocatalytic activity [8,9].

In this study, ZrO_2_ was used as the semiconductor support upon which to deposit the alloy nanoparticles. ZrO_2_ is a non-toxic semiconductor material with high thermal stability and strong chemical resistance [10,11]. High band gap energy and low solar energy conversion rate, which makes pristine ZrO_2_ a low-efficiency photocatalyst, hence, there are some methods to modify the surface to make it a good candidate for photocatalytic reactions. However, the wide bandgap of ZrO_2_ is partly what makes it a good support for plasmonic nanoparticle-deposited photocatalytic systems, because of its catalytic inactivity towards visible light and large surface area and porosity [6].

A few studies have reported on photocatalytic approaches to the deoxygenation of epoxides. Li et al. found that, under UV irradiation, semiconductor TiO_2_ can convert epoxides into their corresponding alkenes with the support of alcohols [12]. The reaction can be conducted at room temperature in the absence of noble metals but needs UV radiation to achieve high product selectivity. In 2012 Ke et al. conducted research with Au nanoparticles supported on CeO_2_ to drive the deoxygenation reaction under visible light irradiation and found that light radiation reduces the apparent activation energy of the reduction reaction [13]. Also under irradiated conditions, the reaction proceeded via a different mechanism from the thermal reaction. It was proposed that the Au nanoparticles’ plasmonic activity and enhanced electromagnetic near-field assisted the activation of the reactant epoxide bonds after reactant adsorption onto the catalyst surface [13]. No literature reports where alloy nanoparticles assisted deoxygenation of epoxides have been identified to date; hence, in this study, Au-Co alloy nanoparticles were explored as heterogenous photocatalysts for the reaction.

Styrene oxide was used as the model epoxide for this study of photocatalytic deoxygenation and compared to previously reported studies, styrene was the only product observed [13,14]. With the alloy catalysts, styrene glycol was detected as a major product component along with styrene. Styrene glycol is a versatile solvent, a useful stabiliser for enzymes and proteins and can be used as a pharmaceutically active compound [15,16]. Styrene glycol is also a widely used chromatographic solvent and is widely used as a precursor in the synthesis of aromatic compounds and polymers [17]. This study reports a new way to synthesise styrene glycol with a synthetic photocatalytic material.

## 2. Materials and Methods

### 2.1. Materials

Zirconium (IV) oxide (ZrO_2_, <100 nm particle size, TEM), gold (III) chloride hydrate (HAuCl_4_·3H_2_O, 99.999%), sodium borohydride (NaBH_4_, ≥98.0%), cobalt (II) nitrate hexahydrate (Co (NO_3_)_2_·6H_2_O, ≥99.9%). All the chemicals are purchased from Merck Life Science Pty Ltd. (Bayswater, Australia) and used as received.

### 2.2. Catalyst Preparation

Pure 3% Au deposited on ZrO_2_ catalysts, pure 3% Co on ZrO_2_ catalysts, and Au-Co alloy nanoparticles on ZrO_2_ were synthesised by the chemical impregnation method which uses NaBH_4_ as a reducing agent. Generally, the Au_2_Co/ZrO_2_ catalysts were prepared as follows. A weight of 0.5 g of ZrO_2_ powder was dispersed in a mixture of the HAuCl_4_ (5.10 mL, 0.01 M) and Co (NO_3_)_2_ (8.50 mL, 0.01 M) aqueous solutions under magnetic stirring at room temperature. An aqueous solution of lysine (0.5 M) was then added to adjust the pH of the medium to the range of 8–9. Into this solution, freshly prepared NaBH_4_ solution was added dropwise, and the resulting mixture was aged for 24 h. The solid product was then separated by centrifugation at 8000 r.p.m. The recovered solid was washed with water (three times) and ethanol (once) and dried at 60 °C in a vacuum oven for 24 h. The obtained powder was used as a 3% weight total of metal present in the Au_2_Co/ZrO_2_ catalyst for reactions. Catalysts with different Au:Co ratios (2.5: 0.5, 2:1, 1.8:1.2, 1.4:1.6, 1:2, and 0.5:2.5) were prepared using a similar method with different quantities of the HAuCl_4_ and Co (NO_3_)_2_ aqueous solutions.

### 2.3. Catalyst Characterisation

X-ray diffraction (XRD) patterns of the samples were collected using the Bruker D8 Advanced Co/Cr XRD machine with CoKα1 radiation (λ = 1.78897 °A) and with an X-ray generator power of 35.0 kV and 40.0 mA (Bruker AXS, Ettlingen, Germany). UV–vis spectra of the samples were collected using the Cary 27 5000 UV-Vis NIR spectrophotometer in the wavelength range of 200–800 nm (Agilent Technologies, Santa Clara, CA, USA). A Kratos AXIS Supra photoelectron spectrometer (Kratos Analytical Ltd., Manchester, UK) was used to analyse the X-ray photoelectron spectra (XPS) of the samples with a 165 mm hemispherical electron energy analyser, and monochromatic Al Kα radiation (1486.6 eV) at 225 W (15 kV, 15 mA) was the incident radiation source. Raman analysis and surface-enhanced Raman spectroscopic (SERS) studies were carried out using a Qontor Raman Spectrometer (Renishaw, Wotton-under-Edge, UK) over the 100 to 800 nm spectral range. A JEOL 2100 transmission electron microscope (TEM), a JEOL ARM200F NeoARM transmission electron microscope from JEOL—Tokyo, Japan, and a high-resolution transmission electron microscope (HR-TEM) equipped with a Gatan Orius SC1000 CCD camera (Gatan Inc., Pleasanton, CA, USA) and an Oxford X-Max EDS instrument (Oxford Instruments, Abingdon, UK) were used to examine catalyst morphology and particle size and composition with a 200 kV accelerating voltage. An Alpha-P Fourier transform infrared spectrometer (Bruker, Ettlingen, Germany) that was fitted with an ATR accessory was used to investigate bond types in the catalysts, and the elemental composition of the catalysts was detected by a Perkin Elmer 8300DV ICP-OES fitted with an ESI SC-4DX autosampler and a PrepFAST 2 sample handling unit (PerkinElmer, Waltham, MA, USA). The radical formation of the reactions was determined by the Bruker Magnettech ESR5000 electron paramagnetic spectrometer (EPR) (Bruker, Billerica, MA, USA) with 5,5-Dimethyl-1-pyrroline N-oxide (DMPO) as a spin trap agent.

### 2.4. The Procedure for the Photocatalytic Reaction for the Deoxygenation of Styrene Oxide

A mixture of 5 mmol of styrene oxide in 5 mL of isopropanol, 0.5 mL of 0.1 M KOH in isopropanol, and 20 mg of catalyst was added to a 20 mL Pyrex glass reaction vessel, which was then sealed with a rubber septum cap. The reaction mixture was stirred magnetically and irradiated with a Nelson halogen lamp (Newcastle, Australia) that emitted visible light over the wavelength range of 400–800 nm. The light intensity at the sample was measured to be 0.5 W/cm^2^. Dark reactions were conducted using an oil bath placed above a magnetic stirrer to control the reaction temperature. The reaction tube was wrapped with aluminium foil to prevent exposure of the reaction mixture to light. After the reaction time was completed, 0.5 mL aliquots were collected and filtered through a Millipore (Burlington, MA, USA) filter (pore size 0.45 μm) to remove the catalyst particulates. The reaction products were analysed using an Agilent 7820A gas chromatograph through gas chromatography (GC). A 6890N Network GC System with a 5973 Inert Mass Selective Detector was used to determine and analyse the product compositions (Agilent Technologies, Shanghai, China).

## 3. Results and Discussion

### 3.1. Catalyst Characterisation

**Figure 1:**
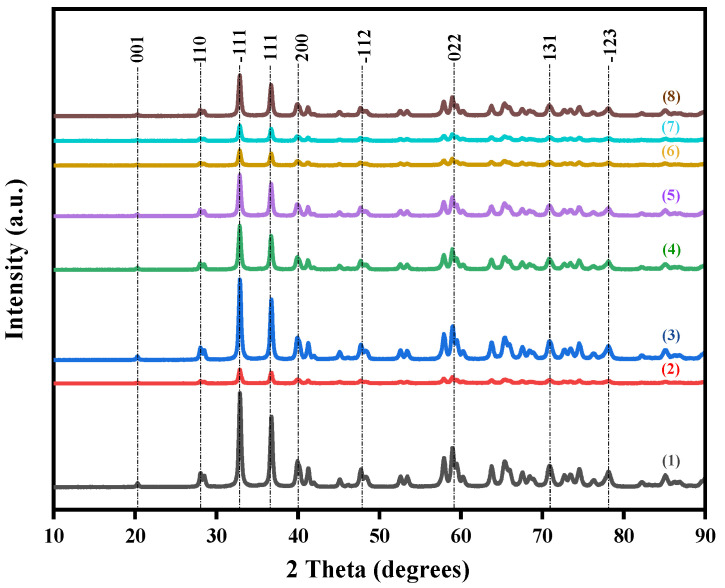
XRD pattern of (1) pristine ZrO_2_, (2) 3% Au/ZrO_2_, (3) Au_2_Co/ZrO_2_, (4) Au_1.8_Co_1.2_/ZrO_2_, (5) Au_1.4_Co_1.6_/ZrO_2_, (6) AuCo_2_/ZrO_2_, (7) Au_0.5_Co_2.5_/ZrO_2_, and (8) Au_1.5_Co_1.5_/ZrO_2_.

Figure 1 presents the XRD patterns of the catalysts prepared with different bimetallic ratios. Irrespective of the Au-Co metal ratios, all samples had the same background XRD pattern, that of monoclinic ZrO_2_. The major zirconia peaks were observed at 2θ values of 20.25°, 27.99°, 32.88°, 36.77°, 39.83°, 40.14°, and 41. 23°, corresponding to the (001), (110), (−111), (111), (200), (020), and (002) planes, respectively, and confirmed by comparison to the standard file PDF 00-065-0728. The absence of Au- or Co-related peaks indicates that alloy nanoparticles were present as small, highly dispersed particles, which do not interfere with the crystal structure of the ZrO_2_ [18]. The variations in peak intensities and peak widths observed in the XRD patterns of the samples with different Au-Co metal loadings indicated that metal loading affects the crystallinity of the catalysts [19]. These differences in crystallinity are often associated with variations in the structural disorders or the defect densities of the material [20,21]. The incorporation of a Au-Co alloy into the ZrO_2_ crystal phase can produce crystal defects which induce a charge imbalance around the dopants and change the stoichiometry of the materials [22]. These changes in defect densities lead to different scattering centres within each sample, which is known to reduce charge recombination by creating localised states within the bandgap that trap electrons and holes which can improve the photocatalytic activity [23]. Comparing the XRD results of the catalyst series, the highest peak intensities occurred in the pristine ZrO_2_ sample, followed by the Au_2_Co/ZrO_2_ catalyst combination. The high intensity and low full-width half-maximum (FWHM) of the peaks indicated higher crystallinity in the nanoparticles. The incorporation of metal nanoparticles lowers the crystallinity of the original ZrO_2_ and the Au_2_Co metal composition allows better preservation of the crystalline structure compared to the other ratios. That could possibly be due to the more favourable interaction or dispersion of Au and Co within the ZrO_2_ matrix [24].

**Figure 2:**
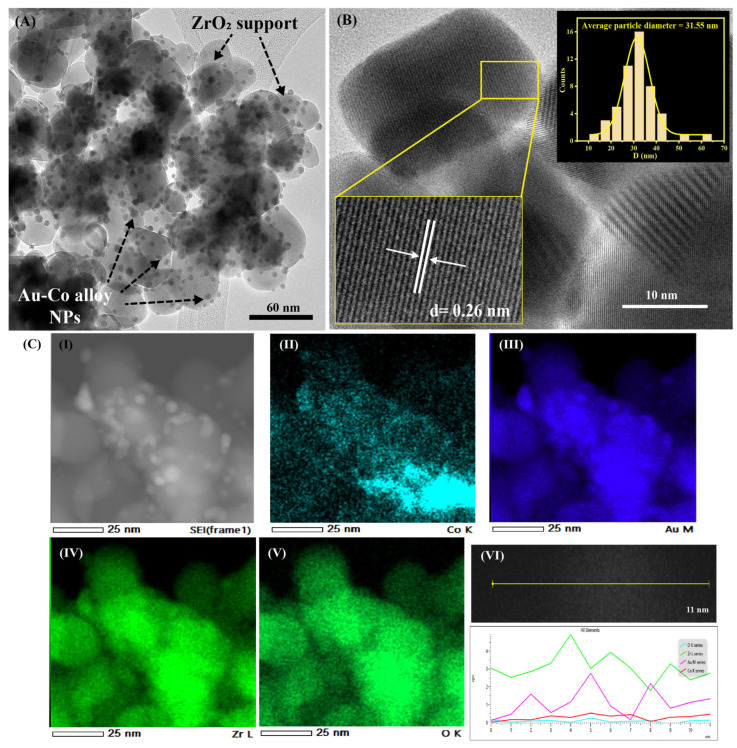
(**A**) A TEM image of Au_2_Co/ZrO_2_ catalyst. (**B**) An HR-TEM image of Au_2_Co/ZrO_2_ catalyst with the average particle diameter distribution curve of the ZrO_2_ support. (**C**) (**I**) A selected area of the Au-Co alloy sample; (**II**) an EDS map of Co K series; (**III**) an EDS map of Au M series; (**IV**) an EDS map of Zr L series; (**V**) an EDS map of O K series; (**VI**) a focused STEM image of the Au-Co alloy/ZrO_2_ particle with an EDS line profile diagram.

The Debye–Scherrer formula was used to calculate the average primary crystallite sizes (D) from the XRD data using the following equation:

D = Kλ/(β cosθ)(1)

where D is the average primary crystallite size, K is equal to 0.9, λ is the wavelength of the X-ray Co Kα radiation (0.178897 nm), β denotes the full width at half maximum (FWHM) of the most intense diffraction peak, and θ is the diffraction angle [10,25]. The calculated ZrO_2_ crystal size for the Au_2_Co/ZrO_2_ catalyst from the XRD data is 15.12 nm, and those for the samples containing other Au:Co metal ratios are provided in the Supporting Information (Table S2). The crystal sizes calculated from XRD data give volume-weighted averages that commonly differ from particle sizes measured directly by TEM, and the differences can be pronounced when polycrystalline aggregates are present [26]. The TEM images of the Au_2_Co/ZrO_2_ catalysts confirmed this; as in Figure 2, large crystalline particles were observed, with an average diameter of 31.55 ± 0.43 nm. Figure 2A clearly highlights the uniform distribution of the Au-Co alloy nanoparticles on the ZrO_2_ support particles. HR-TEM images of the Au_2_Co/ZrO_2_ nanoparticle composites’ interplanar distance confirmed that this was *d* = 2.608 Å which for the ZrO_2_ nanoparticles matched with the (020) plane of monoclinic ZrO_2_.

The uniform distribution of the Au-Co alloy nanoparticles and the formation of alloy nanoparticles on the ZrO_2_ support were characterised in detail using the TEM-EDS technique with an Oxford X-Max 80 mm^2^ silicon drift EDS detector, and the obtained results are represented in Figure 2C. Multiple locations of the sample were analysed with map analysis of the EDS by focusing the beam on a chosen area of the sample, finding the major element components of the sample to be Zr, O, Au, and Co. High-angle annular dark field scanning transmission electron microscopy (HAADF-STEM) images of the sample recognised the Au and ZrO_2_ with contrast differences due to their different atomic numbers. According to the line profile analysis of the TEM EDS in Figure 2C (VI), the Au and Co appeared to combine as alloyed clusters rather than depositing separately on the ZrO_2_.

**Figure 3:**
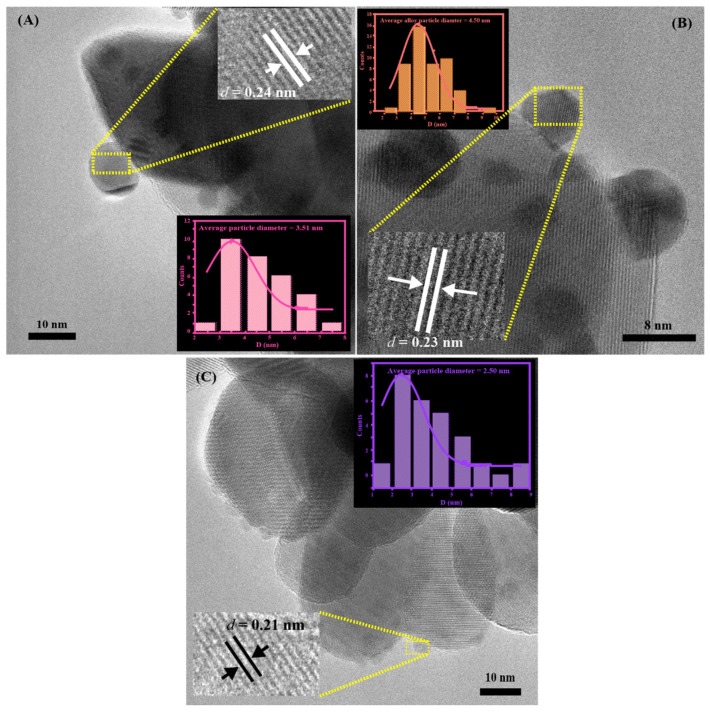
(**A**) HR-TEM of 3% Au/ZrO_2_, (**B**) HR-TEM of Au_2_Co/ZrO_2_, and (**C**) HR-TEM of 3% Co/ZrO_2._

The mean particle diameters of the deposited nanoparticles were determined to be 3.51 ± 0.68 nm for pure Au nanoparticles, 2.50 ± 0.49 nm for pure Co nanoparticles, and 4.50 ± 0.29 nm for Au_2_Co alloy nanoparticles, as shown in Figure 3. The interplanar distance of the pure Au nanoparticle is around 0.24 nm, which corresponds to the (111) plane of gold, and that of 0.21 nm observed in the HR-TEM image corresponds to the (100) plane of hexagonal Co, which is consistent with PDF 01-080-6668 [27]. The average interplanar spacing of the Au_2_Co sample (*d* = 0.23 nm) could not be assigned to any pure metal interplanar spacing of Au (*d* = 0.24 nm) or Co (*d* = 0.21 nm) alone and lies in the intermediate range between those two constituents [27]. The calculated lattice constants from the elemental information agreed with Vegard’s law (d alloy = (2/3 × 0.24) + (1/3 × 0.21)) [28,29]. This gave further confirmation of genuine Au-Co alloy formation in the catalysts.

**Figure 4:**
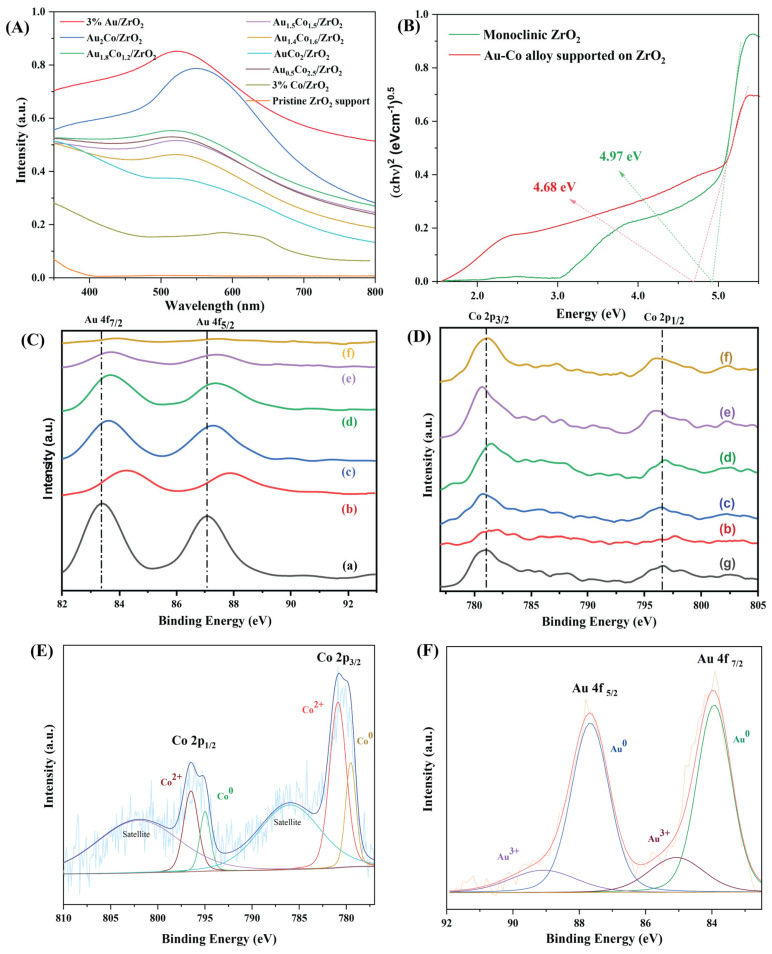
(**A**) UV–vis spectra of the Au-Co alloy-supported ZrO_2_ nanoparticles; (**B**) Tauc plots of the commercial ZrO_2_ and Au_2_Co/ZrO_2_ catalysts used to calculate the optical bandgap; (**C**,**D**) high-resolution XPS of the Au 4*f* states and Co 2*p* states of catalysts loaded with different amounts of Au-Co: (a) Au/ZrO_2_, (b) Au_2_Co/ZrO_2_, (c) Au_1.8_Co_1.2_/ZrO_2_, (d) Au_1.4_Co_1.6_/ZrO_2_, (e) AuCo_2_/ZrO_2_, (f) Au_0.5_Co_2.5_/ZrO_2_, and (g) Co/ZrO_2_; (**E**,**F**) core-level XPS of Co 2*p* and Au 4*f* in the Au-Co/ZrO_2_ catalysts.

ZrO_2_ does not absorb visible light because its band gap is large, around 5 eV. Turning to the UV–vis spectra of Au/ZrO_2_ and the Au-Co alloy/ZrO_2_ catalysts (Figure 4), a strong absorbance peak occurred at 520 nm, which was due to the LSPR effect of the Au nanoparticles. With the inclusion of Co, the LSPR absorption peak dramatically decreased in intensity as the Au content in the nanoparticles decreased. In addition to the dominant LSPR absorption of Au nanoparticles, it is also important to consider that bulk Au can slightly absorb light in the blue and violet regions of the visible spectrum. But, compared to the Au NPs, bulk Au has poor light absorption and catalytic properties [30]. Bulk Au is less effective at generating hot carriers. However, from the nanoscale dispersion of Au and the strong LSPR features observed in the UV–vis spectra, it is clear that the photocatalytic enhancement primarily comes from the plasmon-induced effects of the nanoparticles rather than the bulk absorption.

The chemical impregnation method, besides adhering metal nanoparticles to the surface of the support, can also have the effect of doping the alloy metals within the ZrO_2_ support (at the ZrO_2_ crystal surface). This doping assists in securely attaching the metal nanoparticles to the catalyst support and also enhances their interactions with the ZrO_2_. With dopant metal ion addition, the optical bandgap of the ZrO_2_ decreased due to the introduction of intra-bandgap states into ZrO_2_ [31]. Tauc plot analysis provided a convenient approach to finding the optical bandgaps from the optical absorption spectra of the photocatalyst materials by comparing (αhυ)*^n^* versus hυ, where hυ is the photon energy and α is the Kubelka–Munk function [32,33]. The *n* factor represents the nature of the electron transition, and it has a 1/2 or 2 value for indirect and direct allowed transitions, respectively [33]. Figure 4B gives Tauc plots of the commercial ZrO_2_ and Au_2_Co/ZrO_2_ samples, which confirmed that Au_2_Co/ZrO_2_ has 4.68 eV of reduced bandgap than the 4.97 eV of the original ZrO_2_.

X-ray photoelectron spectroscopy (XPS) is a surface-sensitive technique that is used to identify the elemental compositions and valence states of the elements present in the surface region of the catalysts, which mostly contribute to chemical transformations. The wide XPS scan was performed within an energy range of 0 to 1200 eV and Au, Co, Zr, O, and C were identified as the main elements available on the catalyst surface. High-resolution XPS analysis was then performed to compare the Au 4*f* and Co 2*p* states of the catalysts with different ratios of Au:Co and the results are provided in Figure 4C,D.

The pure Au-containing catalyst showed conclusive evidence of Au 4*f* peaks which occurred at binding energies of 83.9 eV and 87.6 eV and matched with the metallic Au^0^ state, consistent with the previous literature [34,35]. When alloying Au with Co, the Au 4*f* peak slightly shifted to higher binding energies depending on the Au:Co ratio, while Au remains in the reduced state within the alloy system. This shift was more apparent as the Co/Au ratio increased, and this clearly confirmed the formation of Au-Co alloy nanoparticles. Also, as the Co content of the bimetallic samples increased, the binding energy of Co 2*p* shifted to lower energy values compared to those of the monometallic Co sample. The higher electronegativity associated with the Au with respect to the Co leads to an electron transfer between the metals in the alloy. This alters the electron density around the Au and Co atoms, which results in the binding energy shifts seen in the XPS. Furthermore, the Au 4*f* peaks in the Au_2_Co/ZrO_2_ catalysts exhibit a more significant positive shift compared to the other metal compositions. This shift can be attributed to the enhanced electronic interaction between the two metal components in that particular ratio, which may form a strong alloying effect within the catalyst.

Figure 4E,F represent the core-level XPS of Co 2*p* and Au 4*f* of the Au-Co/ZrO_2_ catalysts, respectively. According to the binding energies of the Co 2*p* species, both metallic Co^0^ and Co^2+^ coexist in the catalyst [24,36,37]. In the alloy system, Au is in a zero-valence state, and the surface has some oxidised Au^3+^ species, as exhibited in Figure 4F.

**Figure 5:**
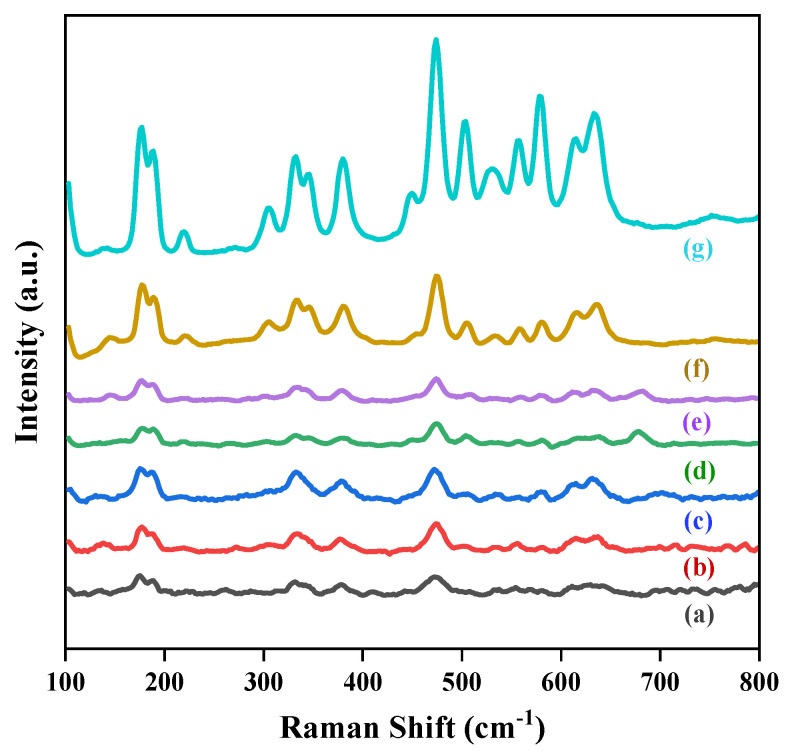
Raman spectra of (a) 3% Au/ZrO_2_, (b) Au_2_Co/ZrO_2_, (c) Au_1.8_Co_1.2_/ZrO_2_, (d) Au_1.4_Co_1.6_/ZrO_2_, (e) AuCo_2_/ZrO_2_, (f) Au_0.5_Co_2.5_/ZrO_2_, and (g) 3% Co/ZrO_2._

The vibrational characteristics of the materials were examined using Raman spectroscopy to investigate possible structural defects, composition, and molecular structure changes in the nanomaterials. Consistent with group theory analysis, there should be 18 vibrational modes (9A_g_ + 9B_g_) of monoclinic ZrO_2_ [38,39]. Out of a total of 18 characteristic peaks, 16 were identified in these catalyst samples at peak positions 177, 189, 221, 305, 332, 347, 382, 448, 475, 503, 528, 535, 558, 579, 614, and 636 cm^−1^ [40]. The Raman spectra of the prepared catalysts are represented in Figure 5, where it can be seen that the intensity of the Raman peaks increased with increases in the Co ratio in the alloy catalysts. Accordingly, the pure Au/ZrO_2_ had the lowest intensity Raman peaks and the highest intensity peaks occurred with the pure Co/ZrO_2_ catalyst. Generally, the intensity of the Raman peaks of a molecule can vary depending on the polarizability of that molecule, the intensity of the source, the concentration of the active group of the molecule, defects, and the crystallite size [41,42]. Hyun et al. concluded in their study that Raman peak intensity varied with the particle diameters of the TiO_2_ nanoparticles [43]. The variation in the Raman bands of PbTiO_3_ with increasing Si content in Si-doped PbTiO_3_ has also been reported by Palkar et al. in their studies [44]. Based on that, the changes in the intensities of the Raman bands in different metal-loaded catalysts can be attributed to the differences in the particle diameters (as in Table S1) and the defect densities which cause changes in the light scattering properties in each catalyst [42].

### 3.2. Catalytic Activity Towards Deoxygenation of Epoxides

#### 3.2.1. Selective Conversion of Styrene Oxide to Styrene with Au/ZrO_2_

**Figure 6:**
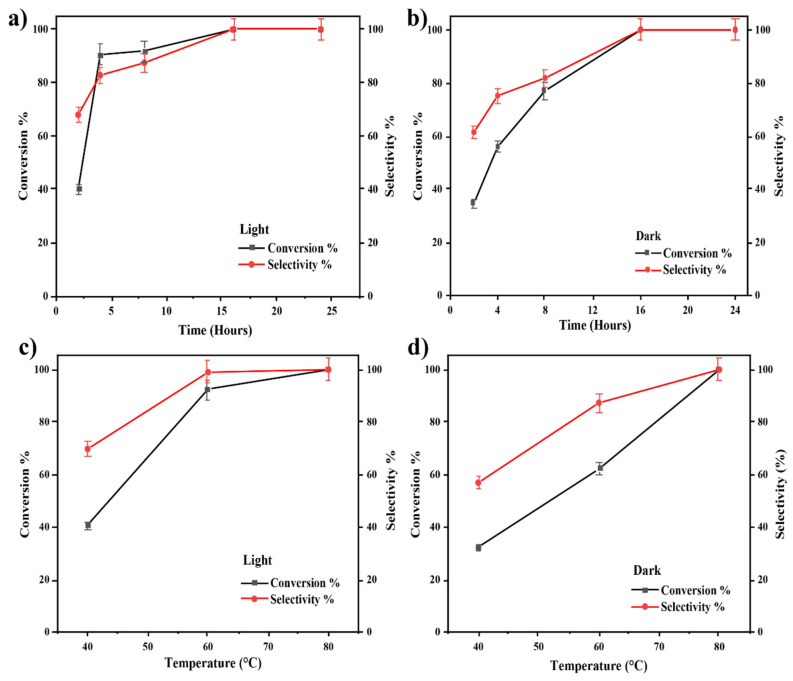
(**a**,**b**) The effect of reaction time on the deoxygenation of epoxide reaction with 3% Au/ZrO_2_ under irradiated conditions and dark conditions. (**c**,**d**) The effect of reaction temperature on the deoxygenation of epoxide reaction with 3% Au/ZrO_2_ under irradiated conditions and dark conditions.

The catalyst of 3 wt% Au-loaded ZrO_2_ support (3% Au/ZrO_2_) was first tested for its catalytic activity in epoxide deoxygenation under visible light irradiation for different time and temperature conditions, based on the previously published work by our group [24,45]. As presented in Figure 6, the conversion and selectivity towards the styrene product were higher under light-irradiated conditions than in dark conditions over the range 40–80 °C. Over this range, the exposure of the reaction mixture to light resulted in a higher reaction conversion. As the reaction temperature was increased, the styrene yield also increased in both dark and light conditions, giving a 99% selectivity of styrene at 60 °C in the case of the irradiated reaction mixture and at 80 °C in dark conditions. Therefore, 60 °C was identified as the preferred temperature for the light reaction which yielded the maximum amount of styrene.

The kinetic study for the reaction performed at 60 °C demonstrated that the exposure of the reaction mixture to light resulted in a higher reaction conversion. The optimum reaction time chosen was 16 h, which is the same as in the study performed by Ke et al., but the reported conversion efficiency in that earlier study was much lower compared to that in this study [13].

**Figure 7:**
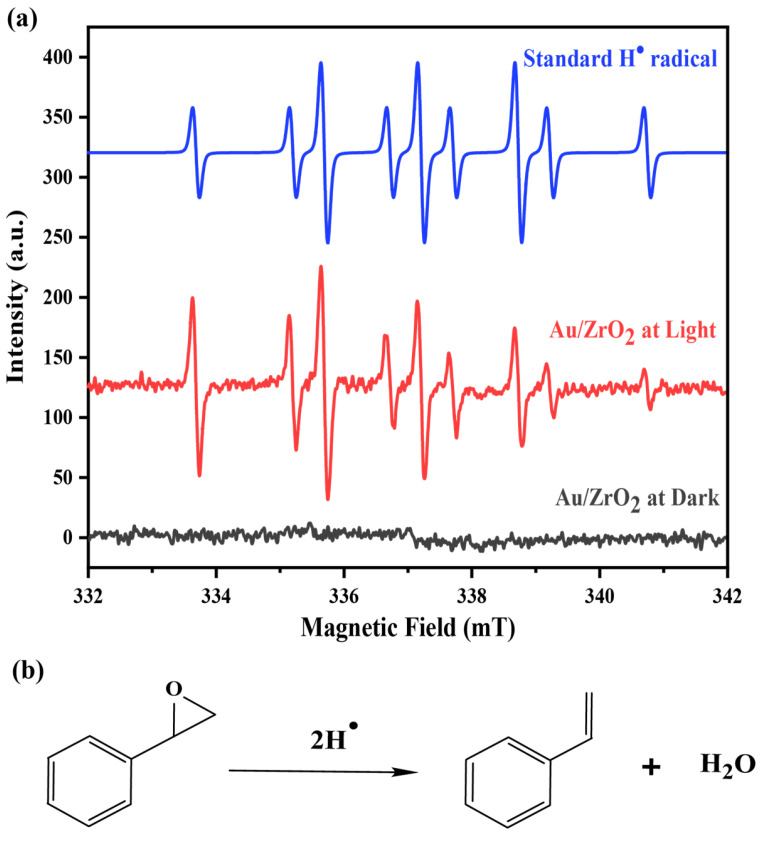
(**a**) EPR spectra of the reaction medium with the Au/ZrO_2_ catalyst under light and dark conditions with DMPO and the simulated EPR spectrum for the reference hydrogen radical. (Simulations of the EPR spectra were performed using SpinFit Liquids software—Version 001 by Bruker (Billerica, MA, USA). A Lorentzian line shape with a line width of 0.432633 G was applied. The Gaussian width was 1.09406 G, and the isotropic orientation of the spin system was assumed). (**b**) A proposed mechanism for the reaction of styrene oxide deoxygenation under light conditions with the Au/ZrO_2_ catalyst.

Therefore, an optimised combination of 3% Au/ZrO_2_ catalyst converted styrene oxide to styrene, achieving a 99% yield of styrene at 60 °C within 16 h under visible light. This was possible because the isopropanol solvent played an important role in the reaction mechanism. It is well-established that isopropanol can act as a hydrogen donor in reduction reactions. In the presence of KOH, plasmonic Au nanoparticles can abstract hydrogen from isopropanol. The importance of the alkaline medium is highlighted by the fact that the reaction’s conversion efficiency was significantly lower in the absence of KOH. Under light irradiation of the semiconductor material, electron and hole pairs are generated and the photogenerated electrons go on to form highly reactive hydrogen radicals (H^•^) by interacting with the abstracted H^+^ ions [46]. The formation of styrene can be facilitated by these generated hydrogen radicals in the medium, as shown in Figure 7b. The EPR spectra for this catalytic reaction in the presence of DMPO spin adducts under dark and under light conditions are provided in Figure 7a, where it is clear that H radical generation only occurred in the light reactions. The EPR spectrum of the reaction with the Au/ZrO_2_ catalyst under illumination in the presence of a DMPO well matches the simulated reference spectrum for the DMPO–hydrogen radical adduct. In the Au/ZrO_2_ EPR spectrum, the peak intensity decreases with increasing magnetic field, likely due to the very short lifetime of the hydrogen radicals formed in the reaction medium. The LSPR effect associated with plasmonic Au nanoparticles is known to generate hot spots, where enhanced local electromagnetic fields occur, and this can assist the activation of epoxide bonds when the reactant molecules are adsorbed onto the catalyst surface [13]. Therefore, the styrene yield is always higher for the irradiated reactions than for the equivalent dark reactions.

**Figure 8:**
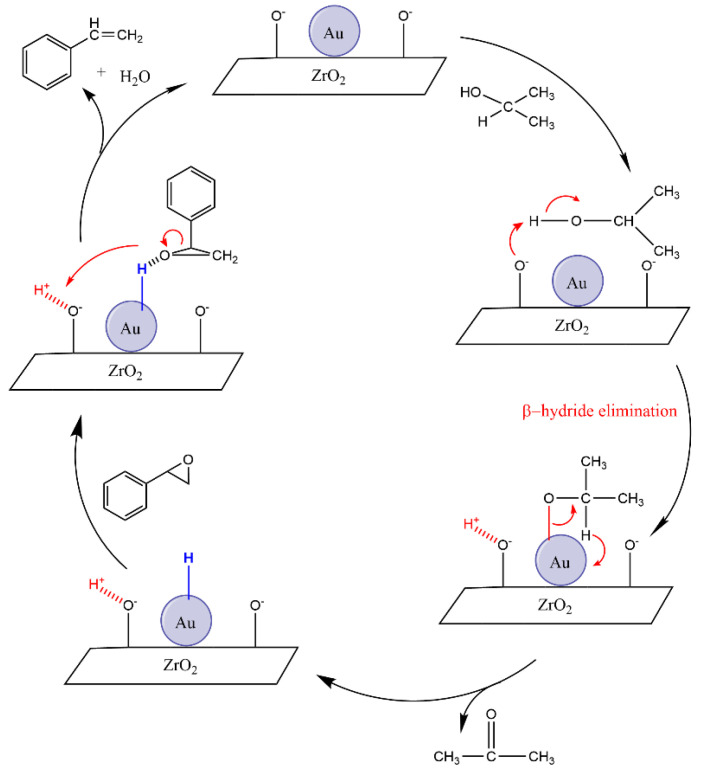
Proposed mechanism for deoxygenation of styrene oxide to styrene with Au/ZrO_2_ catalyst under unirradiated conditions.

Mitsudome et al. and Batra et al. have both reported an ionic mechanism for the deoxygenation reaction within their studies under non-photocatalytic conditions [4,14]. According to this mechanism, the basic sites of the ZrO_2_ support material in the KOH medium and the Au nanoparticles cooperate in this reaction. Initially, the basic sites of the ZrO_2_ support can abstract a proton from adsorbed isopropanol, while the Au nanoparticles interact with the alcoholate species. Then β-hydride elimination leads to the formation of Au-H, which is the active species of the medium. This Au-H species can react with the epoxide group to form an intermediate, which dehydrates to generate styrene, thereby completing the catalytic cycle, even without the light radiation (Figure 8).

#### 3.2.2. Deoxygenation of Epoxides with Au-Co Alloy-Loaded ZrO_2_ Nanoparticles

The formation of metal alloy-supported catalysts can change reaction pathways, facilitate charge separation, and thereby improve photocatalytic activities. Where a plasmonic metal is integrated with another metal, this can significantly change the light absorption properties and photocatalytic activity of these systems. For example, Sarina et al. reported the enhanced catalytic activity of Pd when alloyed with Au to form Au-Pd nanoparticles in the oxidative addition of benzylamine, leading to the selective oxidation of aromatic alcohols and phenol oxidation [9]. In 2016 Liu et al. reported a significant improvement in the product yield of a plasmon-driven CO_2_ reduction reaction with Au-Pd alloy nanoparticles [47]. Also, incorporating Pd atoms into Au nanoparticles has been shown to increase the product yield of the Suzuki–Miyaura cross-coupling reaction under irradiated conditions [48]. Several studies have proven that metal alloy nanoparticles can improve product selectivity by changing the adsorption strength of the organic reactant molecules on the active centres of the substrate [24,49,50]. Therefore, the photoreaction efficiency of the epoxide reduction under different experimental conditions was studied with the Au-Co/ZrO_2_ catalyst under dark and light conditions.

**Figure 9:**
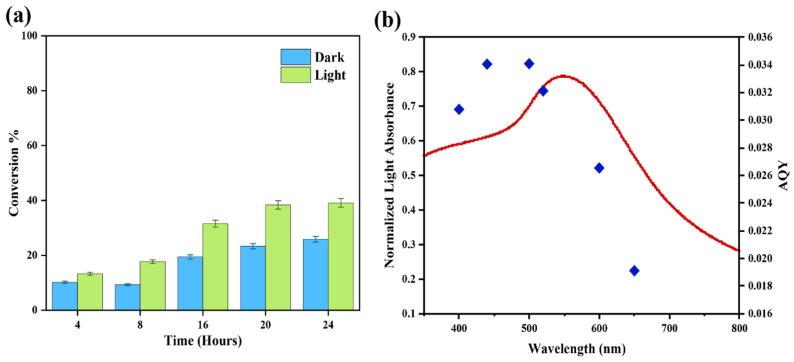
(**a**) The impact of reaction time on the deoxygenation of styrene oxide at 40 °C. (**b**) The action spectrum for the photocatalytic reduction in styrene oxide using the Au_2_Co/ZrO_2_ catalyst.

To assess the catalytic activity of the Au-Co alloy relative to the Au/ZrO_2_ catalyst while minimising the thermal effects, the reaction temperature was maintained at 40 °C. The first observation was that the catalyst of the Au-Co alloy supported on ZrO_2_ (Au_2_Co/ZrO_2_) exhibited a lower conversion efficiency for the reaction, compared to the Au/ZrO_2_ catalyst. However, the reactant conversion was more effective under irradiated conditions than in the dark (Figure 9a). Furthermore, the product selectivity towards styrene was higher under illumination compared to dark conditions. Based on the results of this study, a reaction duration of 24 h was determined to be optimal for the Au-Co alloy supported on ZrO_2_ catalysts (Figure 9a).

Figure 9b displays the action spectrum for the reduction reaction with styrene oxide as the substrate using the Au_2_Co/ZrO_2_ photocatalyst. The action spectrum exhibited close agreement between the wavelength dependence of the photocatalytic rate and the plasmon absorption intensity. Close examination of Figure 9b reveals that the apparent quantum yield (AQY) was greater at slightly shorter wavelengths than the LSPR peak maximum of gold at 520 nm. The maximum AQY value was in the 440 nm to 500 nm range, indicating that the chemical reaction was driven by direct, plasmonic metal light absorption under irradiation. It is possible that the small wavelength offset could be influenced by a photothermal effect which could indirectly increase the reaction activity in that range [50].

**Figure 10:**
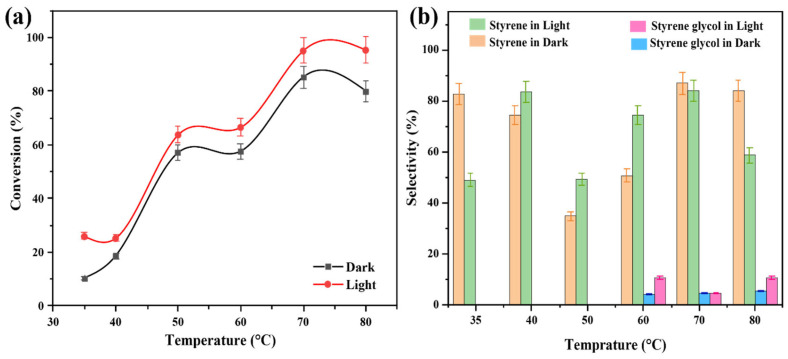
(**a**) The impact of temperature on the conversion efficiency of styrene oxide in the deoxygenation of epoxide reaction with Au_2_Co/ZrO_2_ catalyst. (**b**) The impact of reaction temperature on the product selectivity of the deoxygenation of epoxides with the Au_2_Co/ZrO_2_ catalyst.

The Au-Co/ZrO_2_ catalysts were studied at different temperatures in both light and dark conditions separately. At temperatures of 35 °C, 40 °C, and 50 °C the results in Figure 10a show that styrene was the major reaction product under both light and dark conditions. At temperatures exceeding 60 °C, it was observed that a new product was formed besides styrene, as in Figure 10b. GC-MS analysis identified this new product as styrene glycol (1-phenyl-1,2-ethanediol) under light and dark conditions.

**Figure 11:**
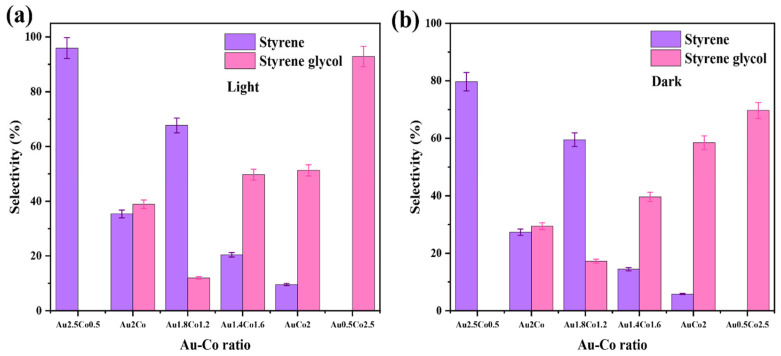
(**a**,**b**) The impact of the Au:Co metal ratio in the catalyst for the product selectivity of the deoxygenation of epoxides reaction at 80 °C under light and dark conditions, respectively.

The styrene glycol product was also observed at 70 °C and 80 °C under both light and dark conditions. These findings indicate that a reaction activation energy barrier is overcome at temperatures higher than 60 °C, which alters the reaction pathway when cobalt is present, resulting in the formation of styrene glycol. Notably, this new product was not observed over the pure Au/ZrO_2_ catalyst under identical conditions, indicating that cobalt is responsible for this alteration. When the alloy’s cobalt content was increased, the styrene glycol product yield increased, with light irradiation also having an effect on product conversion (Figure 11a,b). It was also clearly evident that a lower Au content in the alloy photocatalyst resulted in a lower conversion efficiency, due to decreased plasmonic activity (light absorption at the LSPR frequency) and possibly due to the decreased generation of hot electrons excited to states above the Fermi level of the metal nanoparticles under irradiated conditions [50]. This decrease in plasmon peak absorption as a function of Co content in the Au-Co alloy catalysts is evident in the UV–vis absorption spectra in Figure 4A.

To better understand the influence of thermal energy on the catalytic activity of cobalt itself, a 3% Co/ZrO_2_ catalyst was tested with reactions at 40 °C and 80 °C (Table S3). The Co/ZrO_2_ catalyst did not efficiently produce styrene glycol; this is very different behaviour from the Au-Co alloy catalyst, where the plasmonic activity of the Au component in the alloy led to a high conversion rate to styrene glycol under irradiated conditions. This result confirmed that the plasmonic effect was a considerable factor in driving the reaction towards product formation. To explore the influence of solvent medium and atmosphere on catalysis, the reactions were carried out under different gas systems and solvents, as shown in Tables S4 and S5.

**Figure 12:**
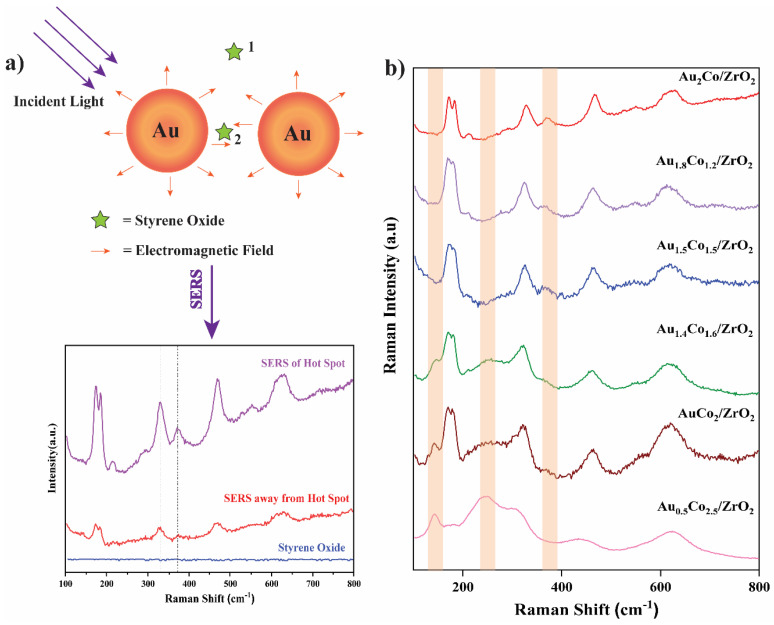
(**a**) A schematic representation of the SERS phenomenon of styrene oxide on the Au_2_Co/ZrO_2_ catalyst and the collected Raman spectrum (Location 1 is the region away from the hot spot and location 2 is the hot spot region). (**b**) The SERS spectra of styrene oxide over the Au-Co alloy NPs supported on ZrO_2._

Since the LSPR effect due to gold was so important to achieve substrate conversion, SERS studies were carried out to confirm the existence of hot spots at the catalyst surface and to study any differences in substrate interactions with the addition of cobalt into the Au-Co alloy. Styrene oxide is converted to pure styrene by the Au/ZrO_2_ catalyst with high selectivity. When Au was alloyed with Co, the reaction path changed, and a new product formed: styrene glycol. Reaction pathway alteration was experimentally tested by the SERS study by analysing the series of catalysts absorbed with styrene oxide molecules under 532 nm of laser with 1800 L/mm grating from the Qontor Raman Spectrometer (Figure 12).

A styrene oxide Raman signal was not detectable in the absence of metal nanoparticles in Figure 12a. However, when styrene oxide molecules adsorbed onto the catalyst, a weak signal corresponding to styrene oxide was detected. When the system was focused on a hot-spot region of the catalyst, a clear SERS enhancement of the styrene oxide Raman signal was observed. For samples with greater Au content in the alloy, the SERS hot-spot density may increase but, as observed in Figure 12a, the Raman signal was highly dependent on the position where the Raman excitation laser beam was focussed.

In the SERS data, some peaks’ intensities changed with the Au-Co alloy content. At around 141.7 cm^−1^ and 248.4 cm^−1^, new peaks appeared in the spectrum when the Co content was increased. These low-frequency Raman peaks often arise from the collective vibrations of a molecule or due to interaction with the SERS substrate [51]. Therefore, the peak around 141.7 cm^−1^ may arise due to the coupling of the aromatic ring vibrations with the glycol group of the styrene glycol [52,53]. The peak appeared around 248.4 cm^−1^ due to the rocking or wagging motions of the hydroxyl groups [53]. Also, the 175.1 cm^−1^, 370.9 cm^−1^, 461.9 cm^−1^, and 549.5 cm^−1^ peaks disappeared with increasing Co content. The peak observed around 549.5 cm^−1^ could be attributed to the C=C-H wagging or twisting vibrational modes of styrene and are progressively diminished with the increasing Co ratio of the catalyst [52]. The catalytic activity of Co in the Au-Co alloy nanoparticle alters the reaction pathway by changing the chemisorption of the styrene oxide molecules onto the catalyst surface, resulting in variations in the SERS peaks. Therefore, with increasing Co ratio, the formation of styrene glycol becomes dominant, while the formation of styrene decreases, which aligns with these SERS results.

**Figure 13:**
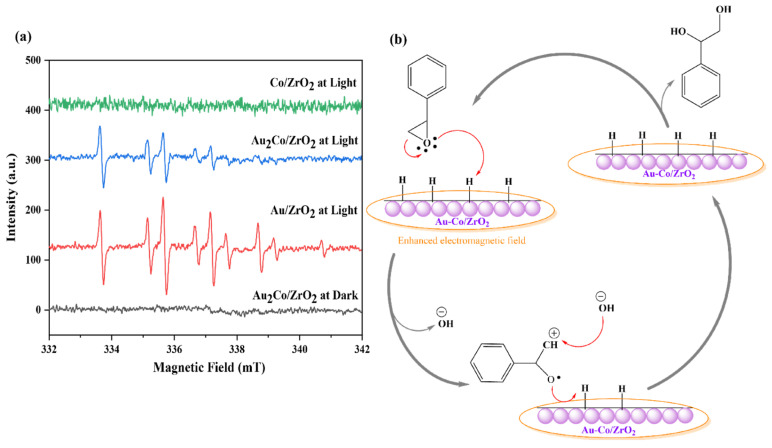
(**a**) EPR Spectra of DMPO adducts detected in the styrene oxide reduction reaction with the Au/ZrO_2_, Au_2_Co/ZrO_2_, and Co/ZrO_2_ catalysts under light and dark conditions. (**b**) The proposed mechanism for styrene glycol formation with the Au-Co/ZrO_2_ catalyst.

EPR studies were conducted to investigate the mechanism of styrene glycol formation in the presence of Co in the catalyst system. The EPR spectra of the DMPO adducts detected during the styrene oxide reduction reaction catalysed by Au_2_Co/ZrO_2_ exhibited significantly lower peak intensities than those observed in the reaction medium catalysed by Au/ZrO_2_. Adding cobalt into the system alters the reaction mechanism and may speed up radical recombination, or hinder radical formation, leading to the disappearance of protonated DMPO EPR peaks and their intensity. When the EPR experiment was conducted using the Co/ZrO_2_ catalyst, no detectable EPR signals were observed, as shown in Figure 13a. This absence of EPR patterns provides strong evidence supporting the ionic mechanism of styrene glycol formation over cobalt-containing catalysts, ruling out the involvement of a radical-based pathway. Furthermore, no radicals were detected under dark conditions, yet the reaction proceeded towards styrene glycol via an ionic mechanism, though with lower efficiency than under light irradiation. Plasmonic Au nanoparticles play a crucial role in radical formation. As the Au content in the catalyst decreases, the radical generation diminishes while shifting the reaction towards an ionic mechanism favouring styrene glycol formation instead of styrene formation.

As shown in Figure 13b, with a small amount of Au in the alloy catalyst, an enhanced electromagnetic field can activate the reactant molecules, concentrating them around the catalyst’s active sites (spillover effect) with light. Then the epoxide moiety can interact with the surface-adsorbed hydrogen of the cobalt catalyst, enabling the hydrogenation reaction to proceed and resulting in the formation of styrene glycol [54,55]. LSPR effects are clearly distinguishable through the formation of hydrogen radicals when gold is present under light irradiation conditions. Hydrogen radicals are absent in dark conditions, as graphically demonstrated by the EPR trapping results given in Figure 7. This is distinct from any potential photothermal contributions. When different catalyst compositions are compared, Co/ZrO_2_ produces exclusively the styrene glycol product in both light and dark conditions (Table S3). Only when gold is included with cobalt does the styrene product form. Gold favours styrene, while cobalt favours styrene glycol. There is a consistent 10–enhanced conversion of reactant when gold is present under light irradiation conditions, as is the case for the Au_2_Co/ZrO_2_ catalysts. This enhancement is consistent over the studied temperature range of 35–80 °C. Styrene production is thus accelerated not only by the presence of gold, but also by light and the LSPR effect through the formation of highly reactive hydrogen radicals, which were observed in radical EPR trapping experiments over the alloy materials under light (but not dark) in Figure 13a.

## 4. Conclusions

In this study, a Au-Co alloy supported on ZrO_2_ nanocatalysts was fabricated using chemical impregnation and the impact of varying Au: Co ratios on their structural and photocatalytic properties was systematically investigated. The chemical and structural features of the catalysts were evaluated with characterisation techniques as XRD, XPS, TEM, EDS analysis, and UV–vis. Photocatalytic test reactions confirmed that 3% pure Au/ZrO_2_ catalysts achieve a 99.99% yield of styrene under light irradiation, while the Au-Co alloy supported on a ZrO_2_ system alters the reaction pathway by forming styrene glycol at elevated temperatures. The SERS studies indicated the influence of the Co molar ratio in alloy catalysts on the differences in the chemisorption of the styrene oxide molecules to the ZrO_2_ support. The EPR studies validated the photocatalytic hydrogen radical generation during the reaction using pure Au/ZrO_2_ catalysts, whereas the alloy system shifted the mechanism towards an ionic pathway. This work demonstrates how the precise tuning of Au:Co metal ratios in the alloy–catalyst system can alter the reaction mechanism and product selectivity by offering new insights into the plasmon-enhanced catalysis.

## Data Availability

Data are contained within the article and Supplementary Materials.

## Supplementary Materials

The following supporting information can be downloaded at: https://www.mdpi.com/article/10.3390/nano15130957/s1. Figure S1: FT-IR analysis of the product mixture with catalysts; Figure S2: (a) HR-TEM of 3% Au/ZrO_2_ and an average particle size distribution curve of 3% Au/ZrO_2_ (b) HR-TEM of 3% Co/ZrO_2_ and an average particle size distribution curve of 3% Co/ZrO_2_ (c) HR-TEM of Au_1.8_Co_1.2_/ZrO_2_ and an average particle size distribution curve of Au_1.8_Co_1.2_/ZrO_2_ (d) HR-TEM of Au_1.4_Co_1.6_/ZrO_2_ and an average particle size distribution curve of Au_1.4_Co_1.6_/ZrO_2_ (e) HR-TEM of AuCo_2_/ZrO_2_ and an average particle size distribution curve of AuCo_2_/ZrO_2_ (f) HR-TEM of Au_0.5_Co_2.5_/ZrO_2_ and an average particle size distribution curve of Au_0.5_Co_2.5_/ZrO_2_; Table S1: Average particle diameters of deposited metal nanoparticles on ZrO_2_ support from TEM analysis; Table S2: Average particle diameters of ZrO_2_ support from the Debye-Scherrer formula; Table S3: The effect of the Co/ZrO_2_ as a catalyst on the Deoxygenation of epoxide reaction under light and dark conditions; Table S4: The effect of the gas in the reaction medium on the Deoxygenation of epoxide reaction under light and dark conditions; Table S5: Product variation of deoxygenation reaction with the solvent medium under light condition; Table S6: Deoxygenation of various epoxides using Au_2_Co/ZrO_2_ catalyst.

## Abbreviations

The following abbreviations are used in this manuscript:

XRD	X-ray Diffraction
XPS	X-ray Photoelectron Spectroscopy
EPR	Electron Paramagnetic Resonance
NPs	Nanoparticles
SERS	Surface-Enhanced Raman Spectroscopy
