# Supplementary material for: Au-Co Alloy Nanoparticles Supported on ZrO2 as an Efficient Photocatalyst for the Deoxygenation of Styrene Oxide
Source: Nanomaterials (Basel). 2025 Jun 20;15(13):957. doi: 10.3390/nano15130957 (PMC12250619; doi:10.3390/nano15130957)
Supplement: Supplementary file 1 [file nanomaterials-15-00957-s001.zip › nanomaterials-3706739-supplementary.pdf]

Supporting Information

Au-Co alloy nanoparticles supported on ZrO_2 as an efficient photocatalyst for the deoxygenation of epoxides

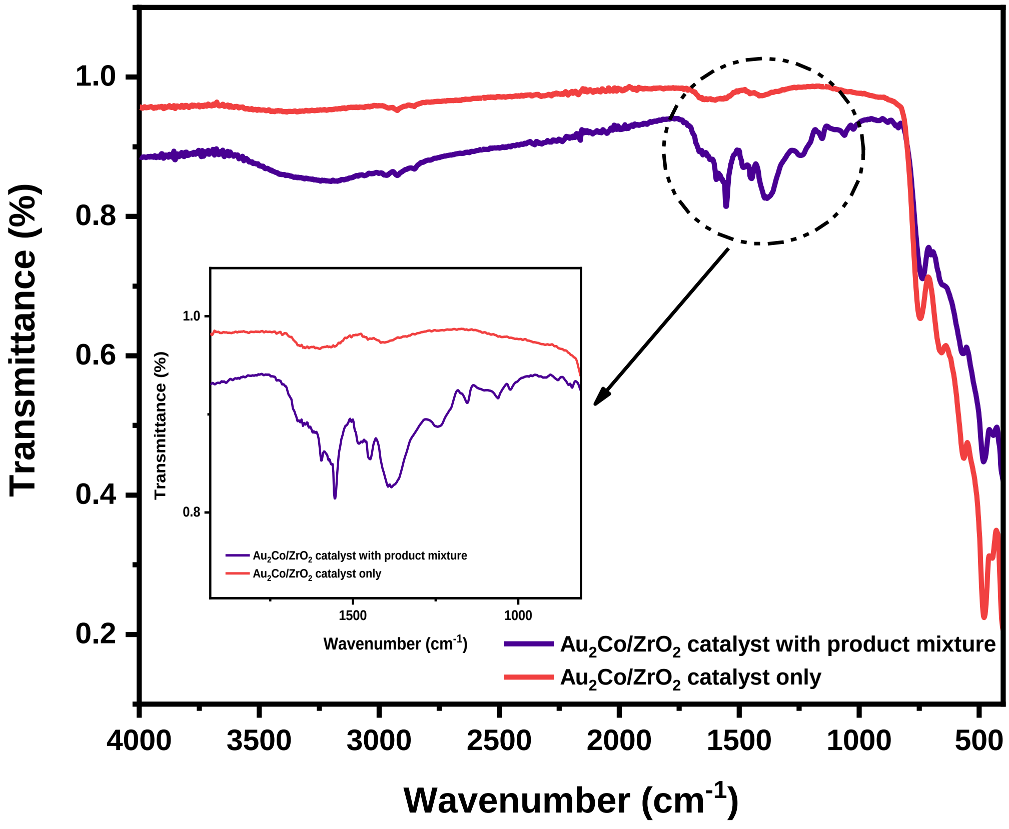

Figure S1. FT-IR analysis of the product mixture with catalysts

FT-IR was used to analyse the bare Au_2Co/ ZrO_2 catalyst and the Au_2Co/ZrO_2 catalyst with the product mixture. As depicted in *Figure S1,* there are new peaks appeared after the reaction due to the newly formed products in the range of 1000 to 1800 cm $^{-1}$. There is a broad peak around the 3240 cm^{-1} that has appeared in the catalyst with products as identified due to the O-H stretching vibration of the alcohol groups of the generated styrene glycol molecules. There is a sharp peak around 1553 cm^{-1} due to the aromatic C=C stretching of the formed styrene and styrene glycol molecules. The peak around 627 cm^{-1} is responsible for the Zr-O vibration mode. Peaks occurred at the positions of

757 cm^{-1} and 500 cm^{-1} due to the symmetric vibrational mode of the Zr-O-Zr bond. Therefore, this analysis confirmed the functional groups of the formed products adsorbed to the catalytic surface.

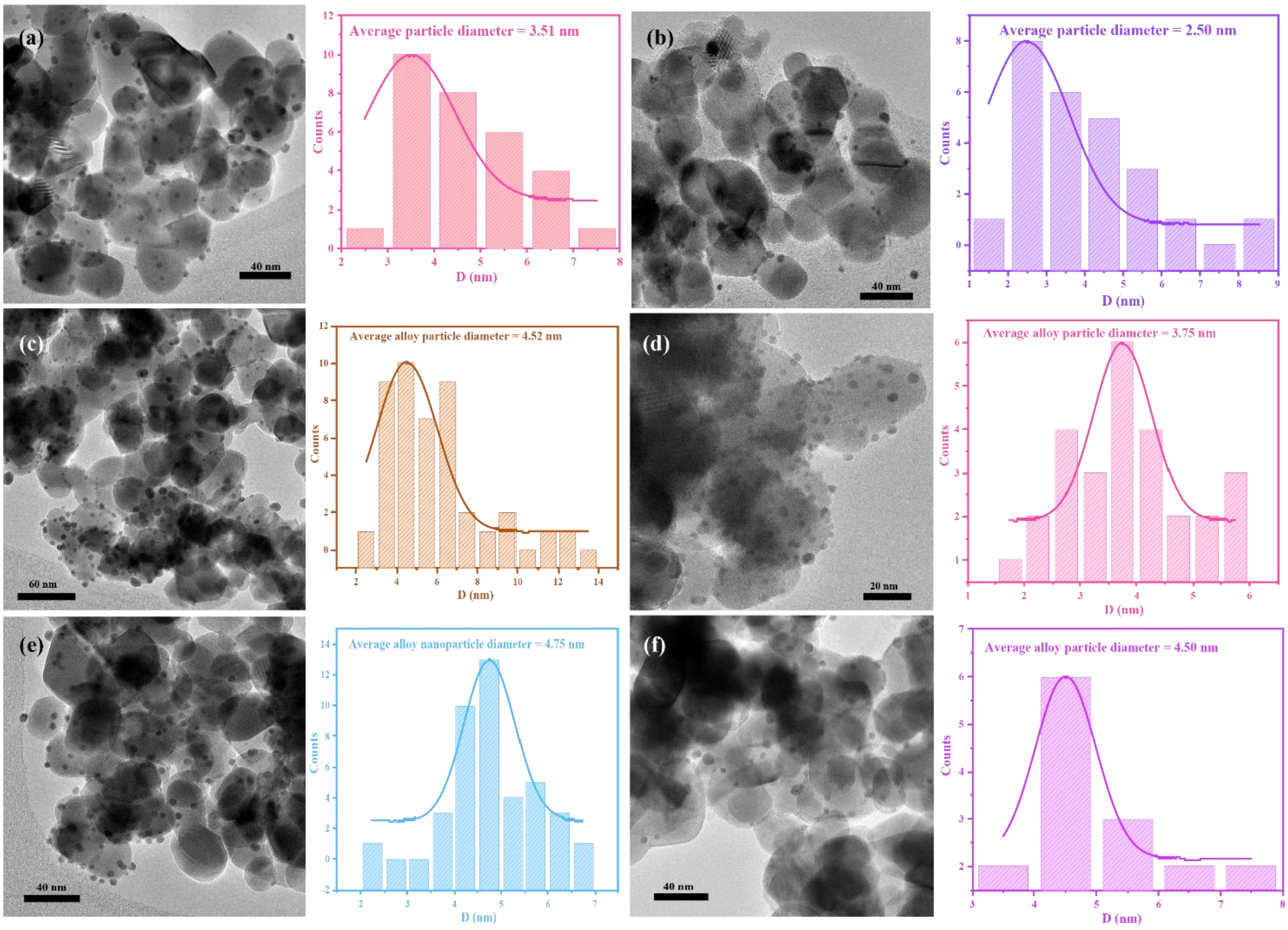

Figure S2. (a) HR-TEM of 3% Au/ZrO_2 and an average particle size distribution curve of 3% Au/ZrO_2 (b) HR-TEM of 3% Co/ZrO_2 and an average particle size distribution curve of 3% Co/ZrO_2 (c) HR-TEM of $Au_{1.8}Co_{1.2}/ZrO_2$ and an average particle size distribution curve of $Au_{1.8}Co_{1.2}/ZrO_2$ (d) HR-TEM of $Au_{1.4}Co_{1.6}/ZrO_2$ and an average particle size distribution curve of $Au_{1.4}Co_{1.6}/ZrO_2$ (e) HR-TEM of $AuCo_2/ZrO_2$ and an average particle size distribution curve of $AuCo_2/ZrO_2$ (f) HR-TEM of $Au_{0.5}Co_{2.5}/ZrO_2$ and an average particle size distribution curve of $Au_{0.5}Co_{2.5}/ZrO_2$

Table S1. Average particle diameters of deposited metal nanoparticles on ZrO_2 support from TEM analysis

**Deposited metal nanoparticle**	**Average particle diameter (nm)**
3% Au	3.51 ± 0.68
Au_2Co	4.50 ± 0.29
$Au_{1.8}Co_{1.2}$	4.52 ± 0.32
$Au_{1.4}Co_{1.6}$	3.75 ± 0.15
$AuCo_2$	4.75 ± 0.15
$Au_{0.5}Co_{2.5}$	4.50 ± 0.27
3% Co	2.50 ± 0.49

Table S2. Average particle diameters of ZrO_2 support from the Debye-Scherrer formula

**Catalyst**	**Average particle diameter (nm)**
3% Au/ZrO_2	9.10
Au_2Co/ZrO_2	26.77
$Au_{1.8}Co_{1.2}/ZrO_2$	22.24
$Au_{1.4}Co_{1.6}/ZrO_2$	15.52
$AuCo_2/ZrO_2$	9.91
$Au_{0.5}Co_{2.5}/ZrO_2$	1.5

Table S3. The effect of the Co/ZrO_2 as a catalyst on the Deoxygenation of epoxide reaction under light and dark conditions

**Entry**	**Temperature (^{0}C)**	**Conversion %**	**Styrene selectivity (%)**	**Styrene glycol selectivity (%)**
**1)**	40	-	-	-

**2)**	80	43.65 (38.82)	-	34.84 (30.15)

Reaction conditions: 20 mg of catalyst, 5 mmol of styrene oxide, 0.5 mL of 0.1 M KOH in isopropanol, Argon atmosphere, 0.5 W/cm^{-1} light intensity, 24 hours, N=2. (Red colour indicates the conversion % in the Dark)

Table S4. The effect of the gas in the reaction medium on the Deoxygenation of epoxide reaction under light and dark conditions

Entry	Gas condition	Conversion %	Styrene Selectivity (%)	Styrene glycol Selectivity (%)
**1)**	Argon	50.16 (23.14)	35.35 (27.31)	38.90 (29.41)
**2)**	Oxygen	-	-	-
**3)**	Air	-	-	-

Reaction conditions: 20 mg of catalyst, 5 mmol of styrene oxide, 0.5 mL of 0.1 M KOH in isopropanol, 0.5 W/cm^{-1} light intensity, 24 hours, 80 ^{0}C temperature, N=2. (Red colour indicates the conversion % in the Dark)

Table S5. Product variation of deoxygenation reaction with the solvent medium under light condition

Entry	Solvent	Major Product	Minor Product
**1)**	Isopropanol	Styrene glycol	Styrene
**2)**	Ethanol	2-Ethoxy-1-phenylethanol	-
**3)**	Propanol	Styrene glycol	Styrene

Reaction conditions: 20 mg of catalyst, 5 mmol of styrene oxide, 0.5 mL of 0.1 M KOH in respective solvent, 0.5 W/cm^{-1} light intensity, 24 hours, 80 ^{0}C temperature.

Table S6. Deoxygenation of various epoxides using Au_2Co/ZrO_2 catalyst

Substrate	Product	Catalyst

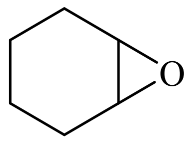	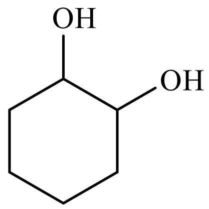	Au_2Co/ZrO_2
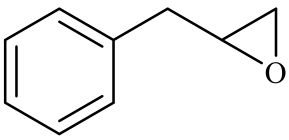	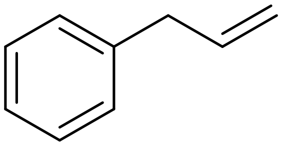	Au_2Co/ZrO_2
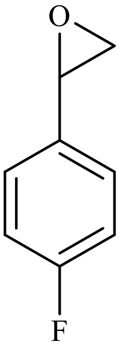	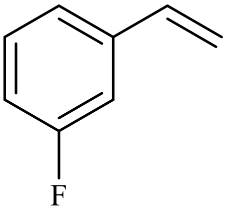 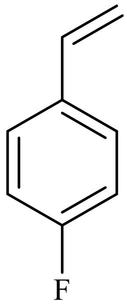	Au_2Co/ZrO_2
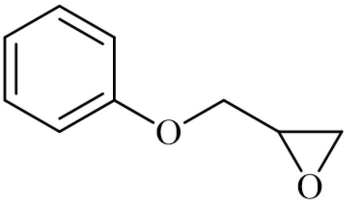	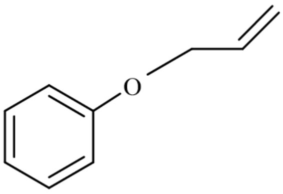 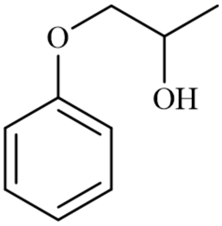 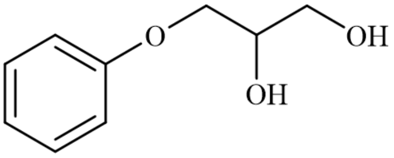	Au_2Co/ZrO_2
